# Rapid ascending aorta stiffening in bicuspid aortic valve on serial cardiovascular magnetic resonance evaluation: comparison with connective tissue disorders

**DOI:** 10.1186/s12968-021-00716-5

**Published:** 2021-02-22

**Authors:** Alejandro Perez-Casares, Audrey Dionne, Kimberlee Gauvreau, Ashwin Prakash

**Affiliations:** 1 Department of Cardiology, Boston Children’s Hospital, Boston, MA USA; 2 Department of Pediatrics, Harvard Medical School, 300 Longwood Avenue, Boston, MA 02115 USA

**Keywords:** Bicuspid aortic valve, Connective tissue disorder, Aortic stiffness, Cardiovascular magnetic resonance

## Abstract

**Background:**

Aortic stiffness has been shown to be abnormal in patients with bicuspid aortic valve (BAV), and is considered a component of the aortopathy associated with this condition. Progressive aortic stiffening associated with aging has been previously described in normal adults. However, it is not known if aging related aortic stiffening occurs at the same rate in BAV patients. We determined the longitudinal rate of decline in segmental distensibility in BAV patients using serial cardiovascular magnetic resonance (CMR) studies, and compared to previously published results from a group of patients with connective tissue disorders (CTD).

**Methods:**

A retrospective review of CMR and clinical data on children and adults with BAV (n = 49, 73% male; 23 ± 11 years) with at least two CMRs (total 98 examinations) over a median follow-up of 4.1 years (range 1–9 years) was performed to measure aortic distensibility at the ascending (AAo) and descending aorta (DAo). Longitudinal changes in aortic stiffness were assessed using linear mixed-effects modeling. The comparison group of CTD patients had a similar age and gender profile (n = 50, 64% male; 20.6 ± 12 years).

**Results:**

Compared to CTD patients, BAV patients had a more distensible AAo early in life but showed a steeper decline in distensibility on serial examinations [mean 10-year decline in AAo distensibility (× 10^−3^ mmHg^−1^) 2.4 in BAV vs 1.3 in CTD, *p *= 0.005]. In contrast, the DAo was more distensible in BAV patients throughout the age spectrum, and DAo distensibility declined with aging at a rate similar to CTD patients [mean 10 year decline in DAo distensibility (× 10^−3^ mmHg^−1^) 0.3 in BAV vs 0.4 in CTD, *p* = 0.58].

**Conclusions:**

On serial CMR measurements, AAo distensibility declined at significantly steeper rate in BAV patients compared to a comparison group with CTDs, while DAo distensibility declined at similar rates in both groups. These findings offer new mechanistic insights into the differing pathogenesis of the aortopathy seen in BAV and CTD patients.

## Background

Bicuspid aortic valve (BAV) is the most common congenital heart defect, with a prevalence of 1–2% in the general population [1]. Many (40–80%) of these patients will develop ascending aortic (AAo) dilation [2, 3]. There is an ongoing debate whether the etiology of this dilatation is due to intrinsic aortic wall abnormalities or abnormal flow dynamics related to valve abnormality, or both [4, 5]. Although often benign, the aortopathy in BAV is progressive and can lead to dissection and death [3, 6]. Increased aortic stiffness has been reported in BAV patients by several investigators and this is considered to be an important component of the aortopathy associated with BAV [7, 8]. Progressive aortic stiffening is associated with aging in normal adults, and in patients with a connective tissue disorder (CTD) [9]. However, it is not known if aging related aortic stiffening is accelerated in BAV patients. In order to improve our mechanistic understanding of the aortopathy associated with BAV, we determined the longitudinal rate of change in segmental aortic stiffness in BAV patients using serial cardiovascular magnetic resonance (CMR), and compared it with patients with CTDs.

## Methods

A retrospective study of existing clinical data at Boston Children’s Hospital from January 2005 through March 2019 was performed. The Department of Cardiology’s Scientific Review Committee and the Boston Children’s Hospital’s Committee on Clinical Investigation gave permission for a retrospective chart review.

### Subjects

Children and adults with BAV with least 2 available clinically indicated CMR examinations (minimum interval between examinations 1 year) were included. Patients with associated complex congenital heart disease (including coarctation of the aorta), a significant shunt lesion, a genetic syndrome, or a CTD were excluded. Patients with unicuspid aortic valve are considered a morphological subset of patients with BAV, sharing complications such us valvar dysfunction and AAo dilation, and were therefore included. We compared the longitudinal changes in aortic stiffness in BAV patients to a group of patients with CTD. Longitudinal changes in aortic stiffness in this CTD group were analyzed using identical techniques and have been previously published by our laboratory [10]. Demographic, clinical and surgical data were abstracted from the hospital’s electronic medical record.

### CMR imaging and analysis

CMR was performed for clinical indication using a commercially available whole-body scanner (Achieva; Philips Healthcare, Best, The Netherlands). In young patients who could not cooperate with breathing instructions, the examination was performed under general anesthesia. Brachial artery blood pressure was measured in the right arm before each examination in the supine position using commercial oscillometric blood pressure recorders. Electrocardiogram (ECG)- gated 2 dimensional cine balanced steady-state free precession (bSSFP) imaging of the left ventricular outflow tract in 2 orthogonal planes was performed that were then used to plan a stack of cine bSSFP images in the short axis of the AAo and the descending aorta (DAo) as previously described [11]. Sievers classification was used to describe the type of BAV [12]. Cine bSSFP images were analyzed at 2 locations (AAo and thoracic DAo) to calculate parameters of stiffness as previously reported [11]. At each location, cross-sectional area was measured by a single observer using manual planimetry at both peak systole and end-diastole. Images were cross-referenced with 2 long axis planes to select the appropriate short-axis slice perpendicular to the aorta. Aortic stiffness was assessed using the following parameters as previously described [11]:

$$Strain=\frac{\mathrm{Systolic}\;\mathrm{area}-\mathrm{Diastolic}\;\mathrm{area}}{\mathrm{Diastolic}\;\mathrm{area}}$$

$$Distensibility(AD)=\frac{\mathrm{Strain}}{\mathrm{Brachial}\;\mathrm{pulse}\;\mathrm{pressure}}$$

$$\beta stiffness\;index=\frac{\ln(systolic\;blood\;pressure/diastolic\;blood\;pressure)}{\mathrm{Strain}}$$

Measurements were performed using commercially available software (cvi^42^ version 5.10, Circle Cardiovascular Imaging Inc. Calgary, Alberta, Canada). Height and weight were measured at each CMR examination and body surface area (BSA) was calculated using the Haycock formula [13]. The rate of AAo dilation was calculated as the change in the BSA-adjusted systolic cross-sectional area between the 2 CMR examinations. The severity of aortic regurgitation was categorized semi-quantitatively on phase-contrast CMR flow measurements, whenever possible. The severity of aortic stenosis was categorized semi-quantitatively on echocardiography performed within 1 year of the CMR examination, when available.

### Statistical analysis

The longitudinal change in aortic stiffness parameters was evaluated through linear mixed-effects modeling and expressed as mean change over 10 years with 95% confidence intervals. Differences in slopes between males and females and other patient subgroups were evaluated using interaction terms. Spearman correlation coefficients were used to assess associations between change in stiffness parameter over time and size of the AAo. Baseline patient characteristics were compared between BAV and CTD patients using Fisher’s exact test and the two-sample t test. For all analyses, a p value less than 0.05 was considered to be statistically significant. Statistical analysis was performed using Stata (version 13.0; Stata Corporation, College Station, Texas, USA).

## Results

### Subjects

Characteristics of BAV subjects and the comparison group of CTD patients are summarized in Table 1. The study population with BAV consisted predominantly of young and middle-age adults with a median age of 18 years, but 14/49 (29%) were children < 18 years of age. A total of 98 CMR examinations were analyzed in 49 patients (2 examinations per patient, median duration between examinations 4.1 years, range 1.0–9.4 years). Consistent with prior studies in patients with BAV, there was male preponderance (73%). Valve stenosis or regurgitation was common, but mild or moderate in severity in a majority of patients. A minority of patients were receiving a β-blocker or an angiotensin converting enzyme inhibitor. The results of the comparison group of CTD patients have been previously reported. The median duration between examinations in the CTD group was 3.9 years (range 1.0 to 13.2). The BAV and CTD groups were similar in their age and gender distribution. The BAV group patients had higher prevalence of aortic stenosis or regurgitation. Rates of β-blocker and angiotensin receptor blocker use were similar among groups.

**Table 1:** Patient characteristics

Parameter	BAV (n = 49)	CTD (n = 50)	p value
Age at first CMR (years)	23.0 ± 11	20.6 ± 12	0.30
Male gender	36 (73%)	32 (64%)	0.26
Type of BAV		N/A	N/A
Left–right	22 (45%)		
Right-non	11 (22%)		
Left-non	1 (2%)		
Unicuspid	15 (31%)		
Type of CTD	N/A		N/A
Marfan syndrome		26 (52%)	
Nonspecific CTD		14 (28%)	
Loeys-Dietz syndrome		7 (14%)	
Ehlers-Danlos syndrome		3 (6%)	
Aortic regurgitation			< 0.001
None/trivial	12 (25%)	47 (94%)	
Mild	30 (63%)	3 (6%)	
Moderate	5 (10%)	0	
Severe	1 (2%)	0	
Not available	1 (2%)	0 (0%)	
Aortic stenosis			< 0.001
Mild or less	23 (50%)	50 (100%)	
Moderate	22 (48%)	0	
Severe	1 (2%)	0	
Not available	2 (4%)	0	
β blocker and/or ARB use	30 (61%)	24 (48%)	0.22

*ARB* denotes angiotensin receptor blocker, *BAV* bicuspid aortic valve, *CTD* connective tissue disorders. The severity of aortic regurgitation and stenosis was assessed by echocardiography

### Longitudinal change in aortic stiffness in BAV

**Fig. 1:**
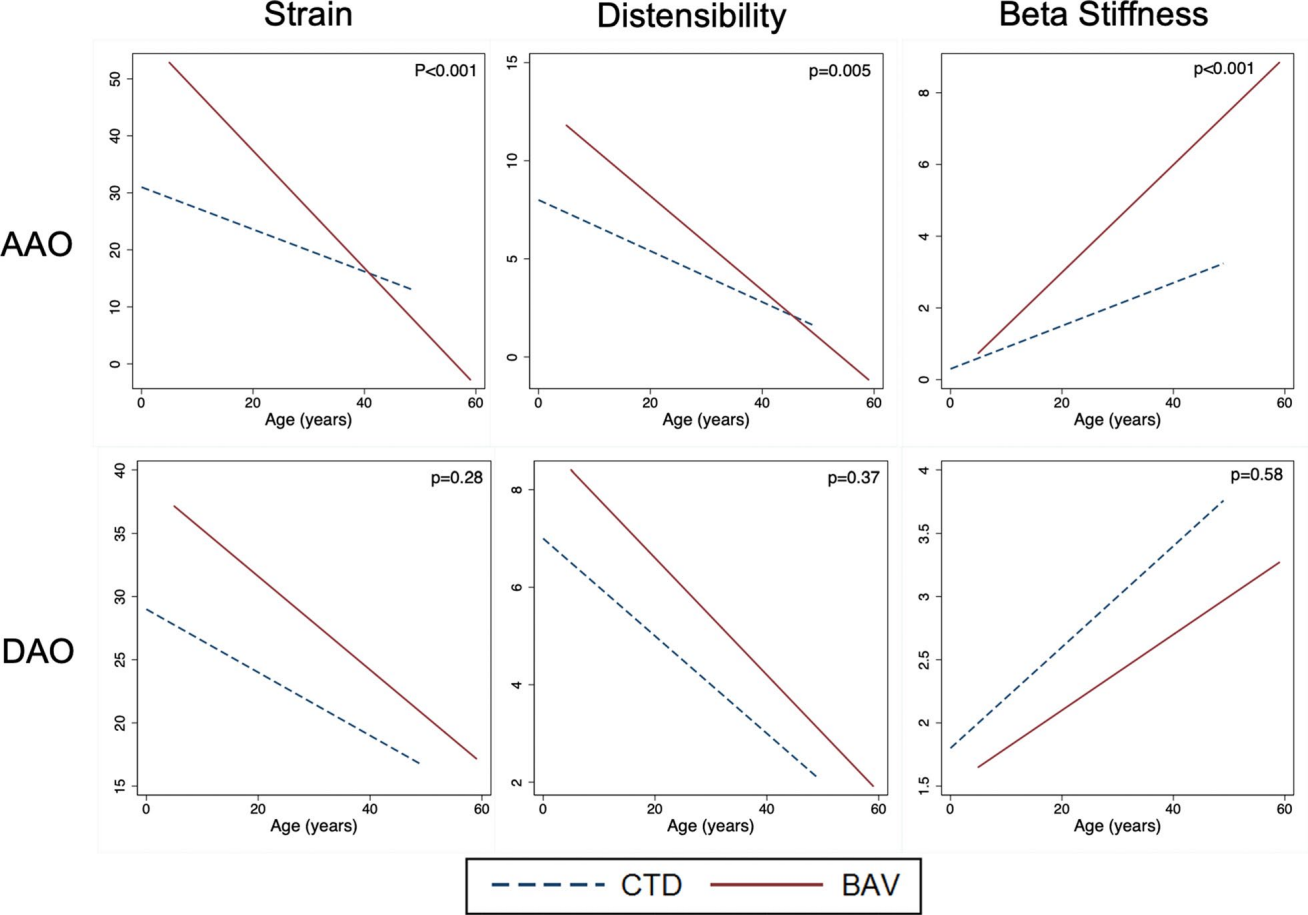
Comparison of the rate of change in aortic stiffness parameters in bicuspid aortic valve (BAV) and connective tissue disorder (CTD) patients at the ascending aorta (AAo) and descending aorta (DAo). Each trendline represents the average change in the stiffness parameters over serial CMR examinations

**Fig. 2:**
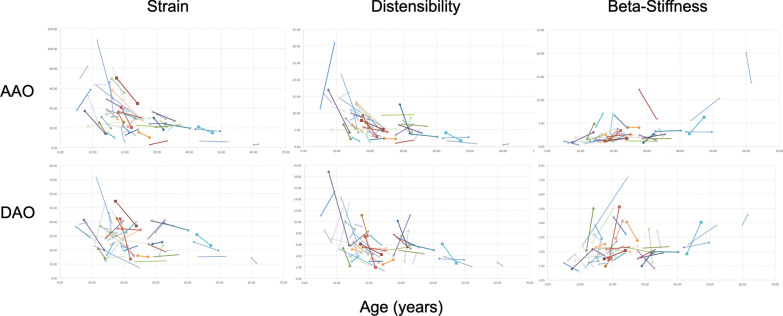
Longitidianal changes in aortic stiffness parameters in individual patients with BAV. Baseline and follow-up measurements in each patient are connected by a straight line. Each patient is depicted using a different colored line

The results of linear mixed-effects modeling to determine the longitudinal rate of change in stiffness parameters are summarized in Table 2 and Fig. 1. Patient-level longitudinal changes are depicted in Fig. 2. Both the AAo and DAo became stiffer with aging, with progressive decline in aortic strain and distensibility, and increase in the β-stiffness index. However, the rate aortic stiffening was steeper at the AAo. We also explored non-linear models and found that they did not provide an improved fit compared to the linear models.

**Table 2:** Comparison of serial change in aortic stiffness in BAV and CTD

Parameter	BAV Mean 10 year change (n = 49)	CTD Mean 10 year change (n = 50)*	*p* value
Ascending aorta (AAo)			
Strain (%)	− 10.3 (− 13.7, − 6.9)	− 3.7 (− 5.3, − 2.0)	< 0.001
Distensibility (× 10^−3^ mmHg^−1^)	− 2.4 (− 3.2, − 1.7)	− 1.3 (− 1.8, − 0.9)	0.005
β− stiffness index	1.5 (1.0, 2.0)	0.6 (0.1, 0.2)	< 0.001
Descending thoracic aorta (DAo)			
Strain (%)	− 3.7 (− 5.8, − 1.5)	− 2.5 (− 3.7, − 1.3)	0.28
Distensibility (× 10^−3^ mmHg^−1^)	− 1.2 (− 1.8, − 0.7)	− 1.0 (− 1.3, − 0.6)	0.37
β-stiffness index	0.3 (0.1, 0.6)	0.4 (0.2, 0.6)	0.58

*Data on CTD patients is derived from previously published data from our laboratory [10]

For each stiffness parameter, the rate of longitudinal change did not vary significantly by baseline severity of aortic stenosis or regurgitation, or by type of BAV. There were no gender differences in the rate of longitudinal change in strain or distensibility at the AAo or DAo. However, the rate of increase in the β-stiffness index at the AAo was faster in females (mean 10-year change 2.4 vs 1.0 in males).

### Comparison with CTD

Detailed results from the CTD group have been previously published [10]. Comparisons between the BAV and CTD groups are presented in Table 2 and Fig. 1. As expected, there was progressive aortic stiffening with increasing age at both the AAo and DAo. However, as seen in Fig. 1, the BAV patients started out with a more distensible AAo earlier in life, but showed a steeper rate of AAo stiffening compared to the CTD patients, resulting in a stiffer AAo at older ages. As seen in Table 2, the mean 10-year rate of change in all the stiffness parameters at the AAo was statistically different between the two groups. In contrast, at the DAo, the BAV patients continued to show a more distensible DAo compared to the CTD group throughout the studied age spectrum and there was no statistically significant difference in the rate of change in stiffness parameters.

## Discussion

In serial CMR analyses of children and young adults with BAV and CTD, aortic strain and distensibility progressively decreased with age. Compared to the CTD group, BAV patients had more distensible AAo early in life but showed a steeper decline in distensibility on serial examinations. In contrast, the DAo was more distensible in BAV patients throughout the age spectrum, and DAo distensibility declined with aging at a rate similar to CTD patients.

### Longitudinal change in aortic stiffness in BAV and comparison with CTD

It has been previously reported that patients with BAV have a stiffer aorta [7, 8]. It is also known that aortic stiffness increases with aging [14, 15]. However, the longitudinal rate of change in aortic stiffness in BAV has not been previously studied. Our results show that aortic stiffness in BAV patients increases at both the AAo and DAo, but the rate of increase is steeper at the AAo, which is the segment often associated with dilation and histopathological wall abnormalities. On comparison with a cohort of patients with CTD-associated aortopathy from our own laboratory, the rate of AAo stiffening was significantly steeper in BAV patients [10]. In contrast, at the DAo, the BAV patients had a more distensible aorta at all ages with a similar age related decline in distensibility, compared to CTD. Notably, although the BAV group had a higher prevalence of aortic valve stenosis and regurgitation, this did not significantly impact the rate of longitudinal change in stiffness parameters.

The rate of aortic stiffening in CTD patients has been previously shown by us to be similar to normal adults [9]. We decided to compare the rate of stiffening in BAV with the previously reported cohort of CTD patients, because these two disorders represent two common etiologies for aortic dilation in children and young adults. The mechanism underlying a faster rate of stiffening of the AAo in BAV are unclear. Both BAV and CTD are associated with histopathologic aortic wall abnormalities that result in stiffening, but there are key differences in their pathogenetic mechanisms. In CTDs, the underlying genetic abnormality results in alterations in the composition of the aortic wall. In BAV, although histologic abnormalities of the aortic wall are noted, an additional role for hemodynamic factors related to increased wall shear stress and abnormal flow profile through the valve has been previously suggested [4, 5, 16, 17]. In this light, it is interesting to note that in this cohort, the baseline distensibility was nearly normal in BAV patients, while CTD had significantly stiff AAos even at baseline. Although this requires further investigation, it could be speculated that increased hemodynamic stress on the AAo wall contributes to the steeper stiffening of the AAo in the BAV patients. These hemodynamic factors are not present in the DAo, where the rate of aortic stiffening is similar in CTD and BAV patients.

### Comparison with other techniques to assess aortic stiffnes and flow

Other techniques for measuring aortic stiffness such as Displacement Encoding with Stimulated Echoes (DENSE) can be used to measure aortic stiffness [18]. This technique allows assessment of regional variations in aortic strain. However, this technique requires a specialized pulse sequence which is not in routine clinical use and was therefore not available in our patients. 4D flow imaging can provide unique insights into the abnormal flow patterns and wall shear stress in patients with BAV [16, 17]. However, these assessments also require the use of a specialized pulse sequence that is not in routine clinical use and was therefore not available for this retrospective analysis.

### Limitations

Several limitations of this work are worth considering. First, it suffers from known limitations of a retrospective design, but these are likely offset at least in part by the uniformity of the imaging protocol. Second, there is potential for referral bias, as CMR was not performed uniformly on all patients with BAV seen at our institution, and this may limit generalizability. Third, a retrospective cohort of healthy children and adults was not available for direct comparison. However, as noted previously, rates of aortic stiffening in healthy adults have been previously reported, and found by us to be similar to patients with CTD [9, 10]. Finally, a significant proportion of patients had aortic valve dysfunction (stenosis or regurgitation) and this could potentially impact measurements of stiffness. However, it is reassuring that in multivariable modeling, the rate of change of stiffness was not impacted by presence and severity of valve dysfunction.

### Conclusions

In longitudinal CMR analyses, children and young adults with BAV had a more distensible AAo compared to CTD patients early in life but showed a steeper decline in distensibility on serial examinations. In contrast, the DAo was more distensible in BAV patients throughout the age spectrum, with a rate of decline similar to CTD patients. These results offer mechanistic insights into the varying pathogenesis of aortopathy in BAV and CTD patients.

## Data Availability

The datasets used and/or analyzed during the current study are available from the corresponding author on reasonable request.

## Abbreviations

AAo: Ascending aorta
BAV: Bicuspid aortic valve
BSA: Body surface area
bSSFP: Balanced steady state free precession
CMR: Cardiovascular magnetic resonance
CTD: Connective tissue disorder
DAo: Descending thoracic aorta
ECG: Electrocardiogram
